# Supplementary material for: Association between Breast Cancer Polygenic Risk Score and Chemotherapy-Induced Febrile Neutropenia: Null Results
Source: Cancers (Basel). 2022 May 31;14(11):2714. doi: 10.3390/cancers14112714 (PMC9179461; doi:10.3390/cancers14112714)
Supplement: Supplementary file 1 [file cancers-14-02714-s001.zip › cancers-1703445-supplementary.pdf]

Supplementary Information

**Item Number**	**Title**	**Main Text Reference**	**Supplementary Information Page Number**
Supplementary Table S1	Description of patients by granulocyte colony-stimulating factor (G-CSF) administration. A total of 441 breast cancer patients who received G-CSF were excluded.	Page 3, 4	2-4
Supplementary Materials and Methods S1	DNA extraction methods, according to the manufacturer's protocols.	Page 4	5
Supplementary Table S2	313 single nucleotide polymorphisms (SNPs) used for calculating breast cancer polygenic risk score (PRS), effect size (odds ratio) and corresponding weights for the overall PRS ($PRS_{overall}$), estrogen receptor (ER)-positive ($PRS_{ER\text{-}pos}$), and ER-negative PRS ($PRS_{ER\text{-}neg}$).	Page 4	6-10
Supplementary Table S3	Association between 313-single nucleotide polymorphism (SNP) breast cancer polygenic risk score and neutropenia-related outcomes in chemotherapy-treated breast cancer patients who did not receive granulocyte colony-stimulating factor (G-CSF).	Page 7	11-15
Supplementary Table S4	Association between 313-single nucleotide polymorphism (SNP) breast cancer polygenic risk score and neutropenia-related outcomes in chemotherapy-treated breast cancer patients who did not receive granulocyte colony-stimulating factor (G-CSF).	Page 9	16

Supplementary Table S1. Description of patients by granulocyte colony-stimulating factor (G-CSF) administration. A total of 441 breast cancer patients who received G-CSF were excluded. [1]Row percentages are presented. [2]Using Kruskal-Wallis test for continuous variables, and Chi-square test for categorical variables to compare patients with no G-CSF administration (reference group) and patients with any GCSF administration (≤30 days before start of chemotherapy, during chemotherapy or ≤30 days after last chemotherapy dose). FNc: febrile neutropenia from initiation of chemotherapy treatment (using taxanes or anthracyclines) to 30 days from last chemotherapy treatment cycle (i.e. within 30 days of last chemotherapy treatment); IQR: Interquartile range; KKH: KK Women's and Children's Hospital; NUH: National University Hospital; SGH: Singapore General Hospital; NCCS: National Cancer Centre Singapore; TTSH: Tan Tock Seng Hospital; HER2: human epidermal growth factor receptor 2; SNP: single nucleotide polymorphism; ER: estrogen receptor.

		G-CSF[1]			
	**Total n=1,596**	**No n=1,155**	**≤30 days before start of chemotherapy n=271**	**During chemotherapy or ≤30 days after last chemotherapy n=170**	***p*-value[2]**
**FNc (n, %)**					
No	1367 (86)	994 (73)	250 (18)	123 (9)	0.500
Yes	229 (14)	161 (70)	21 (9)	47 (21)	
**Neutropenia (n, %)**					
No	1303 (82)	936 (72)	250 (19)	117 (9)	0.350
Yes	293 (18)	219 (75)	21 (7)	53 (18)	
**Demographics**					
**Median age at diagnosis (IQR)**	52 (46 – 59)	52 (46 – 59)	55 (49 – 61)	52 (46 – 59)	<0.001
**Case-type (n, %)**					
Incident	825 (52)	592 (72)	138 (17)	95 (12)	0.611
Prevalent	771 (48)	563 (73)	133 (17)	75 (10)	
**Recruitment Site (n, %)**					
KKH	150 (9)	98 (65)	21 (14)	31 (21)	<0.001
NUH	723 (45)	643 (89)	15 (2)	65 (9)	
SGH & NCCS	267 (17)	261 (98)	2 (1)	4 (1)	
TTSH	456 (29)	153 (34)	233 (51)	70 (15)	
**Year of diagnosis (n, %)**					
Before 2005	91 (6)	89 (98)	1 (1)	1 (1)	<0.001
2005-2010	496 (31)	400 (81)	42 (8)	54 (11)	
2011-2016	1009 (63)	666 (66)	228 (23)	115 (11)	
**Ethnicity (n, %)**					
Chinese	1229 (77)	871 (71)	227 (18)	131 (11)	0.003
Malay	282 (18)	227 (80)	29 (10)	26 (9)	
Indian	85 (5)	57 (67)	15 (18)	13 (15)	
**Body mass index in kg/m^2 (n, %)**					
<20	188 (12)	124 (66)	41 (22)	23 (12)	0.181
20-24	730 (46)	540 (74)	116 (16)	74 (10)	

25-29	467 (29)	337 (72)	78 (17)	52 (11)	
>30	198 (12)	141 (71)	36 (18)	21 (11)	
Unknown	13 (1)	13 (100)	0 (0)	0 (0)	
**Tumour characteristics**					
**Tumour stage (n, %)**					
I	298 (19)	207 (69)	63 (21)	28 (9)	0.013
II	747 (47)	539 (72)	136 (18)	72 (10)	
III	403 (25)	278 (69)	68 (17)	57 (14)	
IV	90 (6)	77 (86)	3 (3)	10 (11)	
Unknown	58 (4)	54 (93)	1 (2)	3 (5)	
**TNM tumour size (n, %)**					
≤20mm	539 (34)	379 (70)	108 (20)	52 (10)	0.799
21 – 50mm	686 (43)	497 (72)	122 (18)	67 (10)	
>50mm	158 (10)	114 (72)	26 (16)	18 (11)	
Attached to chest wall	110 (7)	76 (69)	13 (12)	21 (19)	
Unknown	103 (6)	89 (86)	2 (2)	12 (12)	
**Nodal status (n, %)**					
Positive	687 (43)	474 (69)	140 (20)	73 (11)	0.117
Negative	813 (51)	592 (73)	131 (16)	90 (11)	
Unknown	96 (6)	89 (93)	0 (0)	7 (7)	
**Grade (n, %)**					
Well-differentiated	123 (8)	78 (63)	31 (25)	14 (11)	0.049
Moderately-differentiated	555 (35)	397 (72)	103 (19)	55 (10)	
Poorly-differentiated	805 (50)	595 (74)	126 (16)	84 (10)	
Unknown	113 (7)	85 (75)	11 (10)	17 (15)	
**Estrogen receptor status (n, %)**					
Positive	1047 (66)	759 (72)	185 (18)	103 (10)	0.664
Negative	498 (31)	355 (71)	82 (16)	61 (12)	
Unknown	51 (3)	41 (80)	4 (8)	6 (12)	
**Progesterone receptor status (n, %)**					
Positive	953 (60)	696 (73)	166 (17)	91 (10)	0.310
Negative	590 (37)	416 (71)	101 (17)	73 (12)	
Unknown	53 (3)	43 (81)	4 (8)	6 (11)	
**HER2 status (n, %)**					
Positive	479 (30)	341 (71)	75 (16)	63 (13)	0.587
Negative	922 (58)	642 (70)	182 (20)	98 (11)	

Unknown	195 (12)	172 (88)	14 (7)	9 (5)	
**Proxy subtype (n, %)**					
Luminal A	451 (28)	307 (68)	99 (22)	45 (10)	0.463
Luminal B [HER2-ve)	318 (20)	232 (73)	52 (16)	34 (11)	
Luminal B [HER2+ve)	187 (12)	135 (72)	30 (16)	22 (12)	
HER2-overexpressed	190 (12)	128 (67)	35 (18)	27 (14)	
Triple negative	204 (13)	138 (68)	40 (20)	26 (13)	
Missing	246 (15)	215 (87)	15 (6)	16 (7)	
**313-SNP breast cancer polygenic risk score [median (IQR)]**					
**Overall**	0.355 (-0.007 – 0.720)	0.351 (-0.017 – 0.715)	0.361 (0.058 – 0.764)	0.370 (-0.013 – 0.711)	0.373
**ER-positive**	0.403 (-0.002 – 0.798)	0.399 (-0.010 – 0.788)	0.417 (0.058 – 0.844)	0.375 (0.018 – 0.767)	0.347
**ER-negative**	0.187 (-0.176 – 0.543)	0.184 (-0.179 – 0.545)	0.169 (-0.142 – 0.480)	0.240 (-0.177 – 0.558)	0.866

Supplementary Materials and Methods S1. DNA extraction methods, according to the manufacturer's protocols.

QIAGEN FlexiGene DNA kit (Qiagen, United States, Catalogue number 51206) was used for genomic DNA extraction from buffy coats isolated from whole blood samples according to the manufacturer's protocol. Isolation of DNA from 1–2 ml of buffy coat sample is as follows. Briefly, frozen buffy coat samples were thawed in 37ºC water bath and transferred to a 15ml centrifuge tube. 5 ml of Buffer FG1 (provided in the kit) was added, and inverted to mix. The samples were centrifuged for 5 minutes at 2000 x g in swing-out buckets. Supernatants were discarded before 2 ml of Buffer FG2/20 µl QIAGEN Protease (both were provided in the kit, protease was re-suspended according to manufacturer's recommendations) was added and vortexed immediately until the pellets were homogenized. This was followed by incubation at 65°C for 10 minutes in a water bath. 2 ml of 100% isopropanol was added and mixed thoroughly by inversion until the DNA precipitated. DNA was centrifuged for 3 min at 2000 x g and washed with 2 ml of 70% ethanol. The DNA samples were centrifuged for 3 minutes at 2000 x g and supernatants were discarded. DNA pellets were air-dried and re-suspended with 200-400 µl of Buffer FG3 (provided with the kit) and dissolved by incubating for 1 hour at 65°C in a water bath before DNA quantifications.

DNA Genotek PrepIT-L2P DNA purification kit (DNA Genotek, Canada, Catalogue number PT-L2P-45) was used for genomic DNA extraction from saliva samples collected from Oragene® DNA saliva collection kit (DNA Genotek, Canada, Catalogue number OG-500) according to the manufacturer's protocol. Manual purification of DNA from saliva samples is as follows. Briefly, saliva samples were incubated at 50ºC in an air-incubator for 2 hours before the extraction. 3-4 mL of each sample was transferred to a 15mL centrifuge tube. 120-160 µl of PrepIT-L2P (DNA Genotek, Canada, Catalogue number PT-L2P-45) was added to each sample and vortexed, to precipitate the impurities. Samples were incubated on ice for 10 minutes, followed by centrifugation at room temperature at 3500 x g in swing-out buckets. Supernatants were transferred to new 15mL centrifuge tubes and the impurity pellet was discarded. 3.6-4.8 mL of room temperature 100% ethanol was added to the clear supernatant and samples were inverted to precipitate the DNA. Samples were then centrifuged at room temperature for 10 minutes at 3,500 x g in swing-out buckets to pellet the DNA. Supernatant was discarded and DNA pellets were washed with 1-2 mL of room temperature 70% Ethanol. DNA pellet was re-suspended with 0.2-0.5 mL of 10mM Tris buffer pH8.0 before DNA quantifications.

Supplementary Table S2. 313 single nucleotide polymorphisms (SNPs) used for calculating breast cancer polygenic risk score (PRS), effect size (odds ratio) and corresponding weights for the overall PRS ($PRS_{overall}$), estrogen receptor (ER)-positive ($PRS_{ER\text{-}pos}$), and ER-negative PRS ($PRS_{ER\text{-}neg}$). [1]Weights are the beta values, calculated as the natural logarithm of the odds ratio. PRS: polygenic risk score; $PRS_{overall}$: Overall PRS; $PRS_{ER\text{-}pos}$: ER-positive PRS; $PRS_{ER\text{-}neg}$: ER-negative PRS; No.: Number, corresponding number in Supplementary Table 3 (below) will match to the same SNP; SNP: single nucleotide polymorphism (allele of interest for calculating PRS is included after the underscore); Chr: chromosome number; OR: odds ratio.

				$PRS_{overall}$		$PRS_{ER\text{-}pos}$		$PRS_{ER\text{-}neg}$	
**No.**	**SNP**	**Chr**	**Base-pair Position**	**OR**	**Weight[1]**	**OR**	**Weight[1]**	**OR**	**Weight[1]**
1	rs707475_A	1	7917076	0.960	-0.041	0.961	-0.039	0.945	-0.057
2	rs616488_G	1	10566215	0.943	-0.059	0.960	-0.041	0.895	-0.111
3	rs2992756_C	1	18807339	0.945	-0.056	0.937	-0.065	0.976	-0.025
4	rs4233486_T	1	41380440	1.044	0.043	1.043	0.042	1.040	0.040
5	rs114282204_C	1	41389220	1.168	0.155	1.148	0.138	1.160	0.148
6	rs144105764_T	1	46670206	1.046	0.045	1.061	0.060	1.022	0.022
7	rs56168262_C	1	51467096	1.038	0.037	1.044	0.043	1.004	0.004
8	rs17426269_A	1	88156923	1.051	0.049	1.060	0.058	1.018	0.018
9	rs2151842_A	1	88428199	0.962	-0.039	0.962	-0.038	0.954	-0.047
10	rs612683_T	1	100880328	1.038	0.037	1.036	0.035	1.016	0.016
11	rs56097627_C	1	110198129	1.047	0.046	1.056	0.054	1.027	0.027
12	rs7513707_A	1	114445880	1.064	0.062	1.066	0.064	1.060	0.058
13	rs12406858_C	1	118141492	1.046	0.045	1.043	0.042	1.057	0.055
14	rs637868_C	1	120257110	1.039	0.038	1.044	0.043	1.023	0.023
15	rs11249433_G	1	121280613	1.092	0.088	1.111	0.105	1.021	0.021
16	rs111458676_G	1	121287994	0.935	-0.067	0.922	-0.081	0.989	-0.011
17	rs72127681_CT	1	145604302	0.961	-0.040	0.954	-0.047	0.987	-0.013
18	rs11205303_C	1	149906413	1.056	0.055	1.064	0.063	1.040	0.039
19	rs12091730_A	1	155556971	1.051	0.050	1.062	0.061	1.027	0.026
20	rs139315904_C	1	168171052	0.934	-0.068	0.926	-0.077	0.931	-0.072
21	rs11463354_TA	1	172328767	0.957	-0.044	0.959	-0.042	0.937	-0.065
22	rs35383942_T	1	201437832	1.096	0.092	1.085	0.082	1.074	0.071
23	rs6686987_T	1	202184600	0.994	-0.007	1.013	0.013	0.921	-0.082
24	rs7514172_A	1	203770448	1.051	0.050	1.048	0.047	1.034	0.033
25	rs11268668_TTCTGAAACAGGG	1	204502514	0.968	-0.032	0.998	-0.002	0.874	-0.134
26	rs2785646_A	1	208076291	0.964	-0.037	0.969	-0.031	0.973	-0.028
27	rs2576261_G	1	217053815	1.043	0.042	1.042	0.041	1.048	0.047
28	rs11117758_A	1	217220574	0.957	-0.044	0.955	-0.046	1.003	0.003
29	rs11118563_T	1	220671050	1.043	0.042	1.049	0.048	1.015	0.015
30	rs72755295_G	1	242034263	1.153	0.143	1.164	0.152	1.152	0.141
31	rs78425380_C	2	10138983	1.062	0.060	1.061	0.060	1.047	0.046
32	rs6743383_A	2	19315675	0.967	-0.033	0.977	-0.023	0.945	-0.057
33	rs6725517_G	2	25129473	0.958	-0.043	0.964	-0.036	0.935	-0.067
34	rs12472404_C	2	29179452	0.993	-0.007	1.021	0.021	0.904	-0.101
35	rs4322799_C	2	29615233	0.958	-0.043	0.952	-0.049	0.964	-0.037
36	rs11406722_CT	2	39699510	0.961	-0.040	0.967	-0.034	0.948	-0.054
37	rs6756513_A	2	70172587	0.960	-0.041	0.967	-0.033	0.965	-0.036
38	rs1036759_C	2	88358825	1.048	0.047	1.045	0.044	1.052	0.050
39	rs6746250_G	2	121058254	0.967	-0.033	0.977	-0.023	0.934	-0.068
40	rs17625845_C	2	121089731	0.958	-0.043	0.971	-0.029	0.902	-0.103
41	rs10164550_A	2	121159205	0.957	-0.044	0.951	-0.051	0.984	-0.016
42	rs10179592_C	2	121246568	1.104	0.099	1.096	0.092	1.118	0.111
43	rs17726078_G	2	172974566	0.954	-0.047	0.941	-0.061	0.994	-0.006
44	rs1550622_G	2	174212910	1.061	0.059	1.064	0.062	1.018	0.017
45	rs2356656_T	2	192381934	1.032	0.032	1.018	0.018	1.106	0.101
46	rs10197246_C	2	202204741	0.952	-0.049	0.951	-0.050	0.949	-0.053
47	rs4442975_T	2	217920769	0.877	-0.132	0.858	-0.153	0.943	-0.059
48	2:217955896:GA:G_G	2	217955896	0.817	-0.202	0.790	-0.236	0.946	-0.056
49	rs11693806_G	2	218292158	0.927	-0.076	0.922	-0.081	0.942	-0.060
50	rs3791977_A	2	218714845	0.958	-0.043	0.955	-0.046	0.982	-0.018
51	rs4676356_A	2	241388857	0.884	-0.123	0.875	-0.133	0.841	-0.173
52	rs6762558_G	3	4742251	1.064	0.062	1.063	0.061	1.043	0.042
53	rs552647_A	3	27353716	1.078	0.075	1.086	0.082	1.031	0.031
54	c3_pos27363668_G	3	27388664	1.051	0.050	1.055	0.054	1.030	0.030
55	rs112476261_T	3	29294845	0.880	-0.128	0.885	-0.122	0.742	-0.299
56	rs17838698_T	3	30684907	1.061	0.059	1.068	0.066	1.017	0.017
57	rs56387622_C	3	46888198	0.923	-0.081	0.919	-0.084	0.931	-0.072
58	3:49709912:C:CT_CT	3	49709912	0.964	-0.037	0.965	-0.036	0.930	-0.072
59	rs138866686_AT	3	55970777	0.887	-0.119	0.883	-0.124	0.943	-0.059
60	rs2886671_T	3	59373745	0.961	-0.039	0.957	-0.044	0.961	-0.040
61	rs147250346_TTG	3	63887449	1.067	0.065	1.065	0.063	1.044	0.043
62	rs9825432_G	3	71620370	0.963	-0.037	0.966	-0.034	0.963	-0.038
63	rs13066793_G	3	87037543	0.930	-0.072	0.930	-0.073	0.948	-0.053

64	rs639355_A	3	99403877	0.963	-0.038	0.963	-0.038	0.972	-0.028
65	rs34207738_C	3	141112859	1.057	0.055	1.063	0.061	1.029	0.028
66	rs58058861_A	3	172285237	1.043	0.042	1.051	0.050	0.987	-0.013
67	rs9882792_T	3	189774456	0.953	-0.048	0.954	-0.047	0.956	-0.045
68	rs10012017_T	4	38784633	1.050	0.049	1.051	0.049	1.051	0.050
69	4:84370124:TAA:TA_TA	4	84370124	0.955	-0.046	0.957	-0.044	0.952	-0.049
70	rs17014016_A	4	89240476	1.036	0.035	1.040	0.039	1.026	0.026
71	rs147404208_T	4	92594859	0.960	-0.041	0.963	-0.038	0.965	-0.036
72	rs62331150_T	4	106069013	1.048	0.047	1.061	0.059	1.010	0.010
73	rs147399132_AAT	4	126752992	0.963	-0.038	0.965	-0.036	0.938	-0.064
74	rs56039025_T	4	143467195	0.945	-0.057	0.941	-0.061	0.942	-0.059
75	rs138786872_C	4	151218296	1.040	0.039	1.031	0.031	1.057	0.056
76	rs28436676_A	4	175842495	0.914	-0.090	0.890	-0.116	1.020	0.020
77	rs62334414_A	4	175847436	1.035	0.035	1.055	0.054	0.990	-0.010
78	rs13147907_T	4	187503758	1.036	0.036	1.036	0.035	1.020	0.019
79	rs116095464_C	5	345109	1.088	0.084	1.089	0.086	1.070	0.068
80	rs10069690_T	5	1279790	1.064	0.062	1.033	0.032	1.162	0.150
81	rs3215401_AG	5	1296255	0.947	-0.055	0.959	-0.042	0.900	-0.106
82	rs62329727_C	5	1353077	1.168	0.155	1.170	0.157	1.129	0.121
83	rs4866496_A	5	2777029	1.040	0.039	1.042	0.041	1.023	0.023
84	rs17611291_C	5	16231194	0.958	-0.043	0.955	-0.046	0.960	-0.040
85	rs35130031_T	5	32579616	1.037	0.036	1.040	0.039	1.007	0.007
86	rs58166936_GT	5	44508264	0.889	-0.118	0.882	-0.126	0.900	-0.105
87	rs187108781_G	5	44619502	0.896	-0.110	0.888	-0.119	0.909	-0.096
88	rs4613718_T	5	44649944	1.050	0.049	1.074	0.071	0.974	-0.026
89	rs10941679_G	5	44706498	1.051	0.050	1.067	0.065	0.975	-0.026
90	rs17343002_C	5	44853593	0.967	-0.034	0.978	-0.022	0.925	-0.078
91	rs199562199_CA	5	52679539	1.059	0.057	1.069	0.066	1.043	0.042
92	rs113803968_CT	5	55662540	0.955	-0.046	0.956	-0.045	0.971	-0.030
93	rs889310_T	5	55965167	1.040	0.039	1.039	0.038	1.041	0.041
94	rs16886165_G	5	56023083	1.146	0.137	1.175	0.161	1.071	0.069
95	rs76250845_T	5	56042972	1.090	0.087	1.114	0.108	1.006	0.006
96	rs11949391_C	5	56045081	0.945	-0.056	0.938	-0.064	0.983	-0.017
97	rs113778879_T	5	58241712	0.958	-0.043	0.964	-0.037	0.960	-0.041
98	rs3010266_A	5	71965007	0.960	-0.041	0.956	-0.045	0.976	-0.024
99	rs157557_C	5	73234583	0.964	-0.036	0.952	-0.049	0.990	-0.010
100	rs144028731_G	5	77155397	0.960	-0.041	0.959	-0.042	0.952	-0.049
101	rs34525310_GA	5	79180995	1.033	0.033	1.025	0.025	1.084	0.080
102	rs146817970_T	5	81512947	0.942	-0.060	0.930	-0.073	0.966	-0.034
103	rs332529_A	5	90789470	0.945	-0.056	0.931	-0.071	0.997	-0.003
104	rs17157372_T	5	104300273	0.952	-0.049	0.949	-0.052	0.973	-0.027
105	rs335160_A	5	122478676	0.962	-0.039	0.963	-0.038	0.961	-0.040
106	rs1428387_T	5	122705244	1.099	0.094	1.101	0.096	1.063	0.061
107	rs6860806_G	5	131640536	1.040	0.039	1.048	0.047	1.010	0.010
108	rs6596100_T	5	132407058	0.962	-0.039	0.945	-0.056	0.979	-0.021
109	rs1432679_T	5	158244083	0.935	-0.068	0.938	-0.064	0.937	-0.065
110	rs10074269_C	5	169591460	1.042	0.041	1.051	0.050	1.018	0.018
111	rs6864691_A	5	173358154	1.037	0.036	1.040	0.040	1.035	0.035
112	rs4868701_C	5	176134882	1.037	0.036	1.037	0.037	1.027	0.026
113	rs418053_C	6	13713366	0.946	-0.055	0.940	-0.062	0.985	-0.015
114	rs3819405_T	6	16399557	0.963	-0.037	0.957	-0.044	0.968	-0.032
115	rs12211970_A	6	18783140	1.033	0.033	1.049	0.048	1.003	0.003
116	6:20537845:CA:C_C	6	20537845	0.962	-0.039	0.959	-0.042	0.969	-0.032
117	rs9358466_C	6	21923810	0.968	-0.032	0.957	-0.044	0.997	-0.003
118	rs34196306_C	6	27425644	0.929	-0.074	0.920	-0.084	0.951	-0.051
119	rs111342015_A	6	43227141	0.938	-0.064	0.940	-0.061	0.935	-0.067
120	6:82263549:AAT:A_A	6	82263549	1.049	0.048	1.041	0.041	1.071	0.069
121	rs146519950_C	6	85912194	1.079	0.076	1.059	0.057	1.071	0.068
122	rs73754909_C	6	87803819	1.039	0.038	1.032	0.032	1.070	0.068
123	rs55941023_CT	6	130341728	1.048	0.047	1.044	0.043	1.084	0.080
124	rs2121348_C	6	149595505	0.954	-0.048	0.942	-0.060	0.977	-0.023
125	rs6913578_C	6	151949806	1.073	0.070	1.056	0.054	1.117	0.110
126	rs60954078_G	6	151955914	1.156	0.145	1.122	0.115	1.251	0.224
127	rs57589542_C	6	152022664	1.014	0.014	1.019	0.018	0.983	-0.017
128	rs851984_A	6	152023191	1.065	0.063	1.052	0.051	1.106	0.101
129	rs6904031_T	6	152055978	1.077	0.074	1.065	0.063	1.114	0.108
130	rs910416_T	6	152432902	1.067	0.065	1.054	0.053	1.101	0.096
131	rs9364472_G	6	169006947	0.970	-0.031	0.975	-0.025	0.939	-0.063
132	rs6940159_C	6	170332621	1.038	0.037	1.041	0.040	1.034	0.033
133	rs7971_G	7	21940960	0.954	-0.047	0.960	-0.041	0.944	-0.057
134	rs289997_T	7	25569548	0.953	-0.049	0.953	-0.049	0.934	-0.068
135	rs74765302_A	7	28869017	0.944	-0.057	0.951	-0.050	0.952	-0.049
136	rs13244925_C	7	55192256	0.966	-0.035	0.973	-0.027	0.948	-0.054
137	rs10644978_ATT	7	91459189	1.046	0.045	1.045	0.044	1.050	0.049

138	rs17268829_C	7	94113799	1.046	0.045	1.050	0.049	1.012	0.012
139	rs4439053_A	7	98005235	0.954	-0.047	0.954	-0.047	0.974	-0.027
140	rs111963714_G	7	99948655	1.043	0.042	1.039	0.038	1.051	0.050
141	rs71559437_A	7	101552440	0.945	-0.057	0.928	-0.074	0.976	-0.024
142	rs7800548_C	7	102481842	1.043	0.042	1.041	0.041	1.042	0.041
143	rs12706954_T	7	130656911	0.954	-0.048	0.949	-0.052	0.975	-0.025
144	rs68056147_A	7	130674481	1.042	0.042	1.042	0.041	1.028	0.028
145	rs5887960_C	7	139943702	1.060	0.058	1.069	0.067	1.006	0.006
146	rs62485509_T	7	144048902	0.945	-0.056	0.943	-0.059	0.985	-0.015
147	rs66823261_C	8	170692	1.049	0.048	1.035	0.035	1.110	0.104
148	rs3988353_C	8	17787610	0.963	-0.038	0.964	-0.037	0.971	-0.029
149	rs1028016_G	8	23447496	0.962	-0.039	0.965	-0.036	0.958	-0.043
150	rs310295_A	8	23663653	1.034	0.034	1.046	0.045	1.006	0.006
151	rs9693444_C	8	29509616	0.942	-0.060	0.937	-0.065	0.950	-0.051
152	rs13365225_G	8	36858483	0.927	-0.076	0.931	-0.071	0.904	-0.101
153	rs1511243_G	8	76230943	1.078	0.076	1.085	0.082	1.064	0.062
154	rs72658084_T	8	76333056	1.120	0.113	1.129	0.122	1.092	0.088
155	rs1533366_T	8	76378165	0.962	-0.039	0.959	-0.042	0.974	-0.026
156	rs62517052_C	8	102483100	1.061	0.059	1.076	0.074	1.014	0.014
157	rs12546444_T	8	106358620	0.928	-0.075	0.914	-0.090	0.990	-0.010
158	rs13267382_G	8	117209548	0.959	-0.042	0.955	-0.046	0.960	-0.041
159	rs62526620_G	8	120862186	1.054	0.053	1.062	0.060	1.048	0.047
160	rs35542655_C	8	124563705	1.049	0.048	1.048	0.046	1.052	0.050
161	rs12541094_A	8	124571581	1.035	0.034	1.036	0.035	1.040	0.039
162	rs7842619_G	8	124739913	1.048	0.047	1.040	0.040	1.073	0.071
163	rs35961416_CA	8	128213561	0.958	-0.043	0.954	-0.047	0.961	-0.040
164	rs12550713_G	8	128370949	1.066	0.064	1.085	0.082	1.008	0.008
165	rs10096351_G	8	128372172	1.062	0.060	1.052	0.051	1.059	0.057
166	rs1016578_A	8	129199566	1.063	0.062	1.066	0.064	1.052	0.050
167	rs7830152_G	8	143669254	0.966	-0.035	0.950	-0.052	0.978	-0.022
168	rs10975870_G	9	6880263	1.035	0.035	1.051	0.050	0.992	-0.008
169	rs3057314_C	9	21964882	1.057	0.055	1.048	0.047	1.059	0.058
170	rs17694493_G	9	22041998	1.029	0.029	1.017	0.017	1.095	0.091
171	rs4880038_C	9	36928288	1.025	0.025	1.026	0.026	1.065	0.063
172	rs665889_C	9	87782211	1.037	0.036	1.044	0.043	1.022	0.022
173	rs10120432_C	9	98362587	1.059	0.058	1.064	0.063	1.086	0.083
174	rs60037937_T	9	110303808	1.083	0.080	1.106	0.101	1.013	0.013
175	rs10816625_G	9	110837073	1.123	0.116	1.141	0.132	1.029	0.029
176	rs13294895_T	9	110837176	1.067	0.065	1.084	0.081	0.996	-0.004
177	rs7848334_T	9	110849525	1.015	0.015	1.011	0.011	1.034	0.034
178	rs630965_T	9	110885479	1.092	0.088	1.117	0.111	1.002	0.002
179	rs1895062_G	9	119313486	0.955	-0.046	0.953	-0.048	0.961	-0.040
180	rs3861871_G	9	129424719	0.963	-0.038	0.957	-0.044	0.972	-0.029
181	9:136146597:C:T_T	9	136146597	1.041	0.040	1.041	0.040	1.026	0.025
182	c10_pos5834658_G	10	5794652	1.048	0.047	1.052	0.050	1.039	0.038
183	rs10796139_A	10	13892298	1.038	0.037	1.037	0.036	1.039	0.038
184	rs7072776_G	10	22032942	0.944	-0.058	0.931	-0.072	1.035	0.034
185	10:22477776:ACC:A_A	10	22477776	1.184	0.169	1.182	0.167	1.203	0.185
186	rs10764337_C	10	22861490	1.091	0.087	1.101	0.096	1.020	0.020
187	rs2384736_A	10	38523626	1.041	0.040	1.039	0.038	1.043	0.042
188	rs10995201_G	10	64299890	0.874	-0.134	0.867	-0.143	0.902	-0.103
189	rs6479868_T	10	64819996	1.048	0.047	1.045	0.044	1.041	0.040
190	rs111833376_T	10	71335574	0.960	-0.040	0.960	-0.041	0.947	-0.054
191	rs719338_T	10	80851257	0.923	-0.080	0.914	-0.090	0.957	-0.044
192	rs4980029_G	10	80886726	1.079	0.076	1.081	0.078	1.045	0.044
193	rs140936696_C	10	95292187	0.950	-0.051	0.952	-0.049	0.959	-0.042
194	rs10885405_T	10	114777670	1.048	0.047	1.043	0.042	1.057	0.056
195	rs12250948_C	10	115128491	0.943	-0.059	0.942	-0.060	0.943	-0.059
196	rs9421410_A	10	123095209	0.948	-0.054	0.932	-0.070	1.005	0.005
197	rs45631580_G	10	123340107	1.163	0.151	1.202	0.184	1.005	0.005
198	rs35054928_G	10	123340431	0.786	-0.241	0.747	-0.291	0.968	-0.033
199	rs45631563_T	10	123349324	0.770	-0.261	0.721	-0.327	0.986	-0.014
200	rs7394715_C	11	433617	0.957	-0.044	0.952	-0.049	0.968	-0.032
201	rs6597981_G	11	803017	1.047	0.046	1.041	0.040	1.057	0.056
202	rs4980386_A	11	1895708	0.927	-0.076	0.924	-0.079	0.948	-0.054
203	rs10832963_G	11	18664241	1.047	0.046	1.047	0.046	1.065	0.063
204	rs4472923_T	11	42844441	0.967	-0.034	0.967	-0.033	0.935	-0.067
205	rs10838267_A	11	44368892	1.038	0.037	1.036	0.036	1.021	0.021
206	rs77047825_G	11	46318032	0.928	-0.075	0.933	-0.069	0.934	-0.069
207	rs12287832_A	11	65553492	1.043	0.042	1.045	0.044	1.031	0.030
208	rs10896047_A	11	65572431	0.966	-0.035	0.956	-0.045	0.993	-0.007
209	rs35039974_T	11	69328130	0.959	-0.042	0.948	-0.054	1.014	0.014
210	rs661204_A	11	69330983	1.108	0.102	1.132	0.124	1.018	0.017
211	rs78540526_T	11	69331418	1.195	0.178	1.224	0.202	1.007	0.007

212	rs7125780_G	11	103614438	1.015	0.015	1.003	0.003	1.070	0.068
213	rs199504893_CA	11	108267402	0.998	-0.002	1.014	0.014	0.939	-0.063
214	rs610437_C	11	111696440	0.961	-0.040	0.957	-0.044	0.988	-0.012
215	rs625145_T	11	116727936	0.959	-0.042	0.963	-0.037	0.940	-0.062
216	rs7121616_G	11	122966626	0.962	-0.038	0.963	-0.037	0.953	-0.048
217	rs7939702_G	11	129243417	0.947	-0.054	0.953	-0.048	0.941	-0.061
218	rs11822830_G	11	129461016	1.046	0.045	1.041	0.040	1.061	0.059
219	12:293626:A:G_G	12	293626	1.041	0.040	1.038	0.037	1.040	0.039
220	rs12422552_C	12	14413931	1.050	0.048	1.042	0.041	1.055	0.054
221	rs788458_T	12	28149568	0.940	-0.062	0.951	-0.050	0.934	-0.068
222	rs7297051_T	12	28174817	0.918	-0.086	0.920	-0.083	0.904	-0.101
223	12:28347382:C:T_T	12	28347382	0.949	-0.052	0.956	-0.045	0.954	-0.047
224	12:29140260:G:A_A	12	29140260	1.067	0.065	1.071	0.069	1.078	0.075
225	rs2277339_G	12	57146069	0.944	-0.058	0.943	-0.059	0.939	-0.063
226	12:70798355:A:T_T	12	70798355	1.048	0.047	1.048	0.047	1.015	0.015
227	12:83064195:G:GA_GA	12	83064195	1.069	0.067	1.067	0.064	1.074	0.072
228	12:85004551:C:T_T	12	85004551	1.035	0.035	1.036	0.035	1.036	0.036
229	rs17356907_G	12	96027759	0.917	-0.087	0.919	-0.084	0.939	-0.063
230	12:103097887:C:T_T	12	103097887	1.056	0.055	1.063	0.061	1.015	0.015
231	12:111600134:G:T_T	12	111600134	0.957	-0.044	0.957	-0.044	0.948	-0.054
232	12:115108136:T:C_C	12	115108136	1.048	0.046	1.055	0.053	1.019	0.019
233	12:115796577:A:G_G	12	115796577	0.958	-0.043	0.938	-0.064	0.985	-0.015
234	rs2454399_C	12	115835836	0.922	-0.081	0.907	-0.098	0.985	-0.015
235	12:120832146:C:T_T	12	120832146	1.053	0.052	1.055	0.053	1.054	0.053
236	rs56404467_A	13	32839990	1.043	0.042	1.039	0.039	1.079	0.076
237	rs11571833_T	13	32972626	1.308	0.269	1.260	0.231	1.535	0.428
238	rs9315973_G	13	43501356	1.053	0.052	1.047	0.046	1.102	0.098
239	rs12870942_C	13	73806982	1.035	0.034	1.025	0.025	1.067	0.065
240	rs2181965_G	13	73960952	1.041	0.040	1.037	0.037	1.076	0.073
241	rs34914085_A	14	37128564	0.929	-0.073	0.919	-0.085	0.967	-0.034
242	rs2253012_T	14	37228504	1.040	0.039	1.042	0.041	1.026	0.026
243	rs2588809_C	14	68660428	0.954	-0.047	0.941	-0.061	1.025	0.024
244	rs11624333_C	14	68979835	0.913	-0.091	0.907	-0.097	0.927	-0.076
245	rs11341843_T	14	91751788	1.039	0.038	1.046	0.045	1.009	0.009
246	rs941764_G	14	91841069	1.053	0.051	1.057	0.055	1.032	0.031
247	rs78440108_T	14	93070286	0.944	-0.058	0.949	-0.052	0.943	-0.059
248	rs4983544_G	14	105213978	1.041	0.040	1.036	0.035	1.041	0.040
249	rs187010898_A	15	46680811	0.821	-0.197	0.833	-0.182	0.792	-0.234
250	rs4774565_G	15	50694306	0.959	-0.042	0.961	-0.040	0.962	-0.039
251	rs8042593_A	15	66630569	0.964	-0.037	0.963	-0.038	0.966	-0.034
252	rs35874463_G	15	67457698	1.081	0.078	1.104	0.099	1.014	0.014
253	rs8035987_C	15	75750383	0.960	-0.041	0.959	-0.042	0.959	-0.042
254	rs2290202_T	15	91512267	0.943	-0.059	0.946	-0.056	0.952	-0.049
255	rs144767203_C	15	100905819	0.941	-0.061	0.942	-0.060	0.928	-0.075
256	rs57920543_C	16	4008542	0.968	-0.033	0.982	-0.018	0.915	-0.089
257	rs11076805_A	16	4106788	0.970	-0.030	0.982	-0.018	0.925	-0.078
258	rs12709163_G	16	6963972	1.036	0.035	1.031	0.030	1.084	0.081
259	rs34872983_A	16	10706580	0.929	-0.074	0.927	-0.076	0.952	-0.049
260	rs75753503_T	16	23007047	1.130	0.122	1.146	0.136	1.097	0.093
261	rs35668161_A	16	52538825	1.122	0.115	1.122	0.115	1.096	0.092
262	rs4784227_T	16	52599188	1.113	0.107	1.128	0.120	1.044	0.043
263	rs55872725_T	16	53809123	0.932	-0.070	0.937	-0.065	0.909	-0.096
264	rs6499648_T	16	53861139	0.967	-0.034	0.983	-0.017	0.925	-0.078
265	rs7184573_A	16	53861592	0.967	-0.034	0.966	-0.034	0.970	-0.030
266	rs28539243_A	16	54682064	1.049	0.048	1.057	0.055	1.031	0.030
267	rs7500067_G	16	80648296	1.088	0.084	1.093	0.089	1.048	0.047
268	rs9931038_C	16	85145977	0.979	-0.021	0.996	-0.004	0.931	-0.071
269	rs12449271_C	16	87086492	0.954	-0.047	0.956	-0.045	0.963	-0.037
270	rs79461387_T	17	29168077	0.945	-0.057	0.938	-0.064	0.941	-0.060
271	rs150537328_C	17	39251123	1.083	0.080	1.065	0.063	1.154	0.143
272	rs11296_C	17	40127060	1.018	0.017	0.984	-0.016	1.163	0.151
273	rs17881320_T	17	40485239	0.944	-0.057	0.959	-0.042	0.892	-0.114
274	rs149370081_A	17	40744470	1.223	0.202	1.211	0.192	1.117	0.111
275	rs71363517_CT	17	43212339	1.045	0.044	1.036	0.035	1.049	0.048
276	17:44283858:G:A_A	17	44283858	0.947	-0.054	0.948	-0.053	0.962	-0.038
277	rs2787486_C	17	53209774	0.924	-0.079	0.911	-0.093	0.964	-0.036
278	rs745570_G	17	77781725	0.961	-0.040	0.963	-0.038	0.951	-0.050
279	rs16976596_T	18	11696613	0.963	-0.038	0.972	-0.028	0.910	-0.094
280	rs11665269_T	18	20634253	0.959	-0.042	0.953	-0.049	0.972	-0.028
281	rs1111207_C	18	24125857	1.035	0.035	1.036	0.035	1.023	0.023
282	rs527616_G	18	24337424	1.047	0.046	1.049	0.048	1.029	0.028
283	rs35369219_A	18	24518050	0.942	-0.060	0.920	-0.083	1.006	0.006
284	rs8092192_G	18	25407513	1.041	0.040	1.031	0.031	1.067	0.065
285	rs72931898_A	18	29981526	0.900	-0.106	0.908	-0.096	0.859	-0.152

286	rs9954058_C	18	42411803	0.916	-0.088	0.901	-0.104	0.981	-0.019
287	rs9952980_C	18	42888797	0.947	-0.054	0.943	-0.059	0.963	-0.037
288	rs117922601_T	19	13249921	1.100	0.096	1.097	0.092	1.071	0.069
289	rs56069439_A	19	17393925	1.039	0.038	1.004	0.004	1.184	0.169
290	rs10164323_T	19	18569492	0.931	-0.072	0.925	-0.078	0.939	-0.063
291	rs140702307_CGGGCG	19	19517054	1.045	0.044	1.045	0.044	1.059	0.058
292	rs56681946_C	19	44283031	1.064	0.062	1.062	0.061	1.069	0.067
293	rs4399645_C	19	46166073	0.965	-0.036	0.956	-0.045	0.988	-0.012
294	rs1172821_T	19	55816678	0.965	-0.036	0.963	-0.038	0.966	-0.035
295	rs16991615_A	20	5948227	1.079	0.076	1.079	0.076	1.072	0.069
296	rs1154723_C	20	11379842	1.088	0.084	1.105	0.100	1.078	0.075
297	rs6030585_G	20	41613706	1.032	0.032	1.027	0.027	1.082	0.078
298	rs13039563_A	20	52296849	1.045	0.044	1.055	0.054	1.015	0.014
299	rs2822999_G	21	16364756	1.067	0.065	1.077	0.074	1.033	0.032
300	rs2823130_G	21	16566350	1.061	0.060	1.070	0.068	1.017	0.017
301	rs2403907_A	21	16574455	0.932	-0.071	0.922	-0.081	0.968	-0.033
302	rs4818836_A	21	47762932	1.099	0.095	1.089	0.085	1.093	0.089
303	rs9798754_T	22	19766137	0.964	-0.037	0.958	-0.043	0.978	-0.022
304	rs17879961_G	22	29121087	1.202	0.184	1.325	0.281	0.855	-0.157
305	rs5997390_A	22	29135543	1.068	0.065	1.068	0.066	1.055	0.054
306	rs34134147_T	22	29203724	1.151	0.141	1.196	0.179	1.019	0.019
307	rs132289_G	22	29551872	0.842	-0.172	0.846	-0.167	0.877	-0.132
308	rs373038216_AAAAGAAAG	22	38583315	0.954	-0.047	0.941	-0.061	1.008	0.008
309	rs5750715_A	22	39343916	1.042	0.041	1.033	0.033	1.034	0.033
310	rs66987842_C	22	40904707	1.122	0.115	1.123	0.116	1.128	0.120
311	rs9611990_T	22	43433100	0.942	-0.060	0.943	-0.059	0.950	-0.051
312	rs112855987_A	22	45319953	0.987	-0.013	0.994	-0.006	0.941	-0.061
313	rs28512361_A	22	46283297	1.076	0.074	1.075	0.072	1.104	0.099

Supplementary Table S3. Associations between individual single nucleotide polymorphisms (SNPs) included in the breast cancer polygenic risk score and neutropenia-related outcomes in chemotherapy-treated breast cancer patients who did not receive granulocyte colony-stimulating factor (G-CSF). Controls were chemotherapy-treated breast cancer patients who did not develop neutropenia. [1]Using Fisher's exact test. FNc: febrile neutropenia from initiation of chemotherapy treatment (using taxanes or anthracyclines) to 30 days from last chemotherapy treatment cycle (i.e. within 30 days of last chemotherapy treatment); No.: Number, corresponding number in Supplementary Table 2 (above) will match to the same SNP; SNP: single nucleotide polymorphism (allele of interest measured for allele frequency and analysed for association with neutropenia-related outcomes with Fisher's exact test is included as the first allele after the underscore; second allele after second underscore is the alternative allele); AF: allele frequency of the allele of interest; OR: odds ratio; CI: confidence interval.

		**No neutropenia n=936**	**FNc n=161**			**Neutropenia n=219**		
**No.**	**SNP**	**AF**	**AF**	**OR (95% CI)**	***p*-value[1]**	**AF**	**OR (95% CI)**	***p*-value[1]**
1	rs707475_A_G	0.225	0.205	0.89 (0.66 - 1.19)	0.468	0.203	0.88 (0.68 - 1.13)	0.338
2	rs616488_G_A	0.311	0.326	1.07 (0.83 - 1.38)	0.603	0.329	1.08 (0.87 - 1.35)	0.493
3	rs2992756_T_C	0.175	0.121	0.65 (0.45 - 0.93)	0.015	0.137	0.75 (0.56 - 1.01)	0.055
4	rs4233486_C_T	0.367	0.384	1.08 (0.85 - 1.38)	0.573	0.383	1.07 (0.87 - 1.33)	0.544
5	rs114282204_C_T	0.002	0	-	1.000	0	-	1.000
6	rs144105764_T_TC	0.019	0.012	0.66 (0.23 - 1.87)	0.646	0.011	0.61 (0.24 - 1.56)	0.414
7	rs56168262_C_CT	0.280	0.263	0.92 (0.70 - 1.20)	0.539	0.258	0.89 (0.70 - 1.14)	0.368
8	rs17426269_A_G	0.007	0.009	1.25 (0.36 - 4.37)	0.728	0.007	0.92 (0.26 - 3.20)	1.000
9	rs2151842_A_C	0.119	0.071	0.57 (0.37 - 0.89)	0.013	0.075	0.61 (0.41 - 0.89)	0.009
10	rs612683_A_T	0.500	0.429	0.75 (0.59 - 0.95)	0.022	0.445	0.80 (0.65 - 0.99)	0.044
11	rs56097627_CAAA_C	0.058	0.056	0.97 (0.58 - 1.62)	1.000	0.048	0.82 (0.51 - 1.33)	0.488
12	rs7513707_G_A	0.436	0.469	1.14 (0.90 - 1.45)	0.275	0.475	1.17 (0.95 - 1.44)	0.149
13	rs12406858_C_A	0.415	0.444	1.12 (0.89 - 1.43)	0.359	0.432	1.07 (0.87 - 1.32)	0.554
14	rs637868_T_C	0.087	0.068	0.77 (0.48 - 1.22)	0.328	0.066	0.74 (0.49 - 1.12)	0.178
15	rs11249433_G_A	0.042	0.047	1.12 (0.64 - 1.98)	0.654	0.043	1.04 (0.62 - 1.74)	0.895
16	rs111458676_G_A	0.170	0.177	1.05 (0.77 - 1.43)	0.749	0.169	0.99 (0.75 - 1.31)	1.000
17	rs72127681_CT_C	0.190	0.191	1.01 (0.74 - 1.36)	0.939	0.200	1.07 (0.82 - 1.39)	0.636
18	rs11205303_C_T	0.328	0.311	0.92 (0.71 - 1.19)	0.563	0.317	0.95 (0.76 - 1.19)	0.692
19	rs12091730_G_A	0.376	0.456	1.39 (1.10 - 1.77)	0.008	0.429	1.25 (1.01 - 1.54)	0.044
20	rs139315904_C_CA	0.002	0	-	1.000	0	-	1.000
21	rs11463354_TA_T	0.143	0.140	0.98 (0.69 - 1.38)	1.000	0.151	1.07 (0.80 - 1.44)	0.648
22	rs35383942_T_C	0.006	0.009	1.59 (0.44 - 5.74)	0.446	0.009	1.56 (0.49 - 4.92)	0.504
23	rs6686987_T_C	0.244	0.295	1.30 (1.00 - 1.69)	0.052	0.272	1.16 (0.92 - 1.47)	0.220
24	rs7514172_A_T	0.335	0.311	0.89 (0.69 - 1.15)	0.405	0.320	0.93 (0.75 - 1.17)	0.573
25	rs11268668_T_TTCTGAAACAGGG	0.016	0.019	1.21 (0.50 - 2.94)	0.630	0.021	1.34 (0.63 - 2.84)	0.410
26	rs2785646_A_G	0.026	0.022	0.83 (0.37 - 1.84)	0.848	0.023	0.87 (0.44 - 1.73)	0.866
27	rs2576261_G_T	0.193	0.158	0.79 (0.57 - 1.09)	0.164	0.162	0.81 (0.61 - 1.07)	0.153
28	rs11117758_A_G	0.050	0.075	1.53 (0.96 - 2.44)	0.081	0.068	1.40 (0.91 - 2.14)	0.125
29	rs11118563_T_C	0.310	0.286	0.89 (0.69 - 1.16)	0.396	0.278	0.86 (0.68 - 1.08)	0.205
30	rs72755295_G_A	0.007	0.016	2.26 (0.80 - 6.37)	0.168	0.011	1.65 (0.59 - 4.66)	0.362
31	rs78425380_C_T	0.006	0	-	0.234	0.002	0.35 (0.05 - 2.73)	0.483
32	rs6743383_T_A	0.410	0.404	0.97 (0.76 - 1.24)	0.854	0.425	1.06 (0.86 - 1.31)	0.590
33	rs6725517_G_A	0.397	0.404	1.03 (0.81 - 1.31)	0.853	0.388	0.96 (0.78 - 1.19)	0.745
34	rs12472404_G_C	0.258	0.233	0.88 (0.66 - 1.16)	0.368	0.233	0.88 (0.69 - 1.12)	0.300
35	rs4322799_C_T	0.038	0.040	1.07 (0.58 - 1.95)	0.875	0.034	0.90 (0.51 - 1.59)	0.781
36	rs11406722_C_CT	0.122	0.127	1.05 (0.73 - 1.51)	0.778	0.130	1.08 (0.79 - 1.49)	0.619
37	rs6756513_A_G	0.273	0.317	1.24 (0.96 - 1.60)	0.107	0.317	1.24 (0.99 - 1.55)	0.067
38	rs1036759_C_G	0.280	0.276	0.98 (0.75 - 1.28)	0.946	0.276	0.98 (0.78 - 1.24)	0.906
39	rs6746250_G_A	0.436	0.481	1.20 (0.95 - 1.52)	0.130	0.448	1.05 (0.85 - 1.29)	0.669
40	rs17625845_C_T	0.064	0.062	0.96 (0.59 - 1.57)	1.000	0.071	1.11 (0.74 - 1.67)	0.593
41	rs10164550_A_G	0.145	0.155	1.08 (0.78 - 1.50)	0.670	0.169	1.20 (0.90 - 1.58)	0.234
42	rs10179592_T_C	0.245	0.242	0.98 (0.75 - 1.30)	0.944	0.285	1.23 (0.97 - 1.55)	0.087
43	rs17726078_G_C	0.211	0.162	0.72 (0.52 - 0.99)	0.043	0.180	0.82 (0.63 - 1.07)	0.168
44	rs1550622_A_G	0.013	0.016	1.22 (0.46 - 3.21)	0.604	0.011	0.89 (0.34 - 2.34)	1.000
45	rs2356656_C_T	0.072	0.075	1.04 (0.66 - 1.63)	0.907	0.066	0.91 (0.60 - 1.38)	0.757
46	rs10197246_T_C	0.286	0.276	0.95 (0.73 - 1.24)	0.789	0.267	0.91 (0.72 - 1.15)	0.444
47	rs4442975_G_T	0.151	0.155	1.03 (0.74 - 1.43)	0.867	0.144	0.94 (0.70 - 1.27)	0.766
48	2:217955896:GA:G_G_GA	0.001	0	-	1.000	0	-	1.000
49	rs11693806_G_C	0.314	0.347	1.16 (0.90 - 1.49)	0.270	0.344	1.14 (0.92 - 1.43)	0.232
50	rs3791977_A_G	0.265	0.295	1.16 (0.89 - 1.51)	0.277	0.283	1.10 (0.87 - 1.38)	0.437
51	rs4676356_C_A	0.005	0.003	0.58 (0.07 - 4.55)	1.000	0.002	0.43 (0.05 - 3.34)	0.701
52	rs6762558_G_A	0.113	0.093	0.81 (0.54 - 1.21)	0.335	0.089	0.77 (0.54 - 1.10)	0.171
53	rs552647_A_C	0.292	0.311	1.09 (0.85 - 1.41)	0.508	0.326	1.18 (0.94 - 1.47)	0.164
54	c3_pos27363668_G_C	0.177	0.208	1.22 (0.91 - 1.64)	0.184	0.208	1.22 (0.94 - 1.58)	0.131
55	rs112476261_T_C	0	0	-	1.000	0	-	1.000
56	rs17838698_C_T	0.320	0.317	0.98 (0.76 - 1.27)	0.948	0.317	0.99 (0.79 - 1.23)	0.909
57	rs56387622_C_T	0.140	0.171	1.27 (0.92 - 1.74)	0.145	0.167	1.23 (0.93 - 1.63)	0.153
58	3:49709912:C:CT_CT_C	0.051	0.043	0.85 (0.48 - 1.51)	0.678	0.041	0.80 (0.48 - 1.34)	0.461
59	rs138866686_AT_A	0.002	0.006	2.92 (0.53 - 16.00)	0.216	0.005	2.14 (0.39 - 11.73)	0.319
60	rs2886671_C_T	0.346	0.276	0.72 (0.56 - 0.94)	0.015	0.297	0.80 (0.64 - 1.00)	0.050

61	rs147250346_TTG_T	0.129	0.146	1.15 (0.82 - 1.61)	0.422	0.146	1.15 (0.86 - 1.55)	0.348
62	rs9825432_G_T	0.052	0.031	0.59 (0.30 - 1.14)	0.124	0.034	0.65 (0.37 - 1.13)	0.138
63	rs13066793_G_A	0.011	0.012	1.11 (0.38 - 3.25)	0.778	0.009	0.81 (0.28 - 2.38)	1.000
64	rs639355_A_G	0.448	0.422	0.90 (0.71 - 1.14)	0.396	0.461	1.05 (0.85 - 1.30)	0.631
65	rs34207738_C_CTT	0.061	0.043	0.69 (0.39 - 1.23)	0.248	0.055	0.89 (0.56 - 1.39)	0.656
66	rs58058861_A_G	0.311	0.295	0.93 (0.72 - 1.20)	0.602	0.320	1.04 (0.83 - 1.30)	0.731
67	rs9882792_T_C	0.095	0.088	0.92 (0.60 - 1.39)	0.756	0.087	0.91 (0.63 - 1.32)	0.714
68	rs10012017_T_G	0.432	0.416	0.94 (0.74 - 1.19)	0.626	0.413	0.93 (0.75 - 1.15)	0.520
69	4:84370124:TAA:TA_TAA_TA	0.282	0.311	1.15 (0.89 - 1.48)	0.317	0.308	1.13 (0.90 - 1.42)	0.291
70	rs17014016_A_G	0.022	0.019	0.85 (0.36 - 2.01)	0.837	0.021	0.94 (0.45 - 1.94)	1.000
71	rs147404208_TTCTTTC_T	0.390	0.424	1.15 (0.91 - 1.47)	0.264	0.423	1.15 (0.93 - 1.42)	0.228
72	rs62331150_G_T	0.400	0.388	0.95 (0.75 - 1.22)	0.712	0.390	0.96 (0.78 - 1.19)	0.745
73	rs147399132_AAT_A	0.348	0.311	0.84 (0.65 - 1.09)	0.204	0.306	0.83 (0.66 - 1.03)	0.104
74	rs56039025_T_C	0.039	0.047	1.20 (0.68 - 2.13)	0.538	0.055	1.43 (0.89 - 2.29)	0.145
75	rs138786872_C_CATATTT	0.239	0.236	0.98 (0.74 - 1.30)	0.944	0.233	0.96 (0.75 - 1.23)	0.803
76	rs28436676_A_G	0.259	0.267	1.04 (0.80 - 1.36)	0.783	0.272	1.07 (0.84 - 1.35)	0.587
77	rs62334414_A_C	0.034	0.022	0.64 (0.29 - 1.41)	0.306	0.030	0.88 (0.48 - 1.61)	0.767
78	rs13147907_T_A	0.411	0.332	0.71 (0.56 - 0.92)	0.008	0.340	0.74 (0.59 - 0.92)	0.007
79	rs116095464_C_T	0.011	0.006	0.58 (0.13 - 2.49)	0.760	0.005	0.42 (0.10 - 1.82)	0.409
80	rs10069690_T_C	0.210	0.183	0.84 (0.62 - 1.14)	0.297	0.180	0.83 (0.63 - 1.08)	0.168
81	rs3215401_AG_A	0.414	0.385	0.89 (0.70 - 1.13)	0.358	0.379	0.87 (0.70 - 1.07)	0.195
82	rs62329727_C_T	0	0	-	1.000	0	-	1.000
83	rs4866496_G_A	0.227	0.220	0.96 (0.72 - 1.28)	0.829	0.222	0.97 (0.75 - 1.24)	0.849
84	rs17611291_C_G	0.186	0.189	1.02 (0.76 - 1.38)	0.877	0.169	0.89 (0.68 - 1.17)	0.450
85	rs35130031_T_TCA	0.134	0.115	0.84 (0.58 - 1.21)	0.373	0.119	0.87 (0.63 - 1.20)	0.432
86	rs58166936_GT_G	0.010	0.009	0.92 (0.27 - 3.12)	1.000	0.011	1.13 (0.42 - 3.03)	0.794
87	rs187108781_G_A	0.003	0	-	1.000	0	-	0.591
88	rs4613718_C_T	0.400	0.391	0.96 (0.76 - 1.23)	0.805	0.390	0.96 (0.78 - 1.19)	0.745
89	rs10941679_A_G	0.478	0.450	0.89 (0.71 - 1.13)	0.365	0.448	0.88 (0.72 - 1.09)	0.264
90	rs17343002_C_G	0.036	0.047	1.30 (0.73 - 2.30)	0.346	0.046	1.27 (0.76 - 2.11)	0.335
91	rs199562199_CA_C	0.178	0.188	1.07 (0.79 - 1.45)	0.693	0.197	1.14 (0.87 - 1.48)	0.334
92	rs113803968_CT_C	0.394	0.355	0.85 (0.66 - 1.09)	0.213	0.366	0.89 (0.71 - 1.10)	0.298
93	rs889310_C_T	0.430	0.450	1.09 (0.86 - 1.38)	0.504	0.441	1.04 (0.85 - 1.29)	0.708
94	rs16886165_G_T	0.397	0.419	1.10 (0.86 - 1.39)	0.460	0.432	1.15 (0.93 - 1.42)	0.194
95	rs76250845_T_C	0.119	0.127	1.08 (0.76 - 1.55)	0.643	0.135	1.16 (0.85 - 1.57)	0.372
96	rs11949391_C_T	0.046	0.028	0.60 (0.30 - 1.20)	0.181	0.032	0.69 (0.39 - 1.22)	0.240
97	rs113778879_C_T	0.316	0.338	1.10 (0.86 - 1.42)	0.437	0.318	1.01 (0.81 - 1.26)	0.954
98	rs3010266_A_G	0.158	0.153	0.96 (0.69 - 1.34)	0.868	0.156	0.99 (0.74 - 1.31)	1.000
99	rs157557_C_T	0.394	0.410	1.07 (0.84 - 1.36)	0.622	0.425	1.14 (0.92 - 1.40)	0.255
100	rs144028731_G_GT	0.196	0.211	1.10 (0.82 - 1.47)	0.545	0.194	0.99 (0.76 - 1.28)	0.947
101	rs34525310_GA_G	0.390	0.351	0.85 (0.66 - 1.08)	0.193	0.356	0.87 (0.70 - 1.07)	0.209
102	rs146817970_T_TA	0.006	0.003	0.48 (0.06 - 3.73)	0.706	0.002	0.35 (0.05 - 2.73)	0.483
103	rs332529_A_G	0.449	0.441	0.97 (0.76 - 1.23)	0.808	0.438	0.96 (0.78 - 1.18)	0.709
104	rs17157372_T_G	0.060	0.062	1.04 (0.64 - 1.70)	0.899	0.057	0.95 (0.61 - 1.49)	0.911
105	rs335160_C_A	0.412	0.373	0.85 (0.66 - 1.08)	0.197	0.381	0.88 (0.71 - 1.09)	0.256
106	rs1428387_T_C	0.099	0.068	0.66 (0.42 - 1.05)	0.081	0.068	0.67 (0.45 - 0.99)	0.045
107	rs6860806_G_A	0.264	0.267	1.02 (0.78 - 1.33)	0.891	0.274	1.05 (0.83 - 1.33)	0.674
108	rs6596100_T_C	0.120	0.121	1.01 (0.70 - 1.45)	0.926	0.114	0.94 (0.68 - 1.31)	0.806
109	rs1432679_T_C	0.389	0.398	1.03 (0.81 - 1.32)	0.805	0.397	1.03 (0.84 - 1.28)	0.786
110	rs10074269_C_T	0.500	0.503	1.01 (0.80 - 1.28)	0.952	0.507	1.03 (0.83 - 1.27)	0.832
111	rs6864691_A_G	0.319	0.326	1.03 (0.80 - 1.33)	0.796	0.313	0.97 (0.78 - 1.22)	0.864
112	rs4868701_C_T	0.488	0.466	0.91 (0.72 - 1.16)	0.469	0.477	0.96 (0.78 - 1.18)	0.710
113	rs418053_C_G	0.367	0.370	1.01 (0.79 - 1.29)	0.950	0.388	1.09 (0.88 - 1.35)	0.410
114	rs3819405_T_C	0.331	0.311	0.91 (0.71 - 1.18)	0.520	0.338	1.03 (0.83 - 1.29)	0.778
115	rs12211970_G_A	0.298	0.366	1.36 (1.06 - 1.74)	0.016	0.331	1.17 (0.93 - 1.46)	0.185
116	6:20537845:CA:C_C_CA	0.327	0.345	1.08 (0.84 - 1.39)	0.560	0.345	1.08 (0.87 - 1.35)	0.496
117	rs9358466_C_T	0.309	0.326	1.08 (0.84 - 1.39)	0.558	0.315	1.03 (0.82 - 1.28)	0.819
118	rs34196306_C_G	0	0	-	1.000	0	-	1.000
119	rs111342015_A_G	0.006	0.009	1.46 (0.41 - 5.19)	0.473	0.009	1.43 (0.46 - 4.45)	0.523
120	6:82263549:AAT:A_A_AAT	0.389	0.409	1.08 (0.85 - 1.38)	0.534	0.406	1.07 (0.87 - 1.32)	0.548
121	rs146519950_C_CAA	0.001	0	-	1.000	0	-	1.000
122	rs73754909_C_T	0.264	0.232	0.84 (0.63 - 1.12)	0.257	0.251	0.93 (0.73 - 1.19)	0.622
123	rs55941023_C_CT	0.107	0.099	0.92 (0.62 - 1.36)	0.769	0.114	1.07 (0.77 - 1.49)	0.670
124	rs2121348_C_T	0.392	0.416	1.11 (0.87 - 1.41)	0.423	0.427	1.16 (0.94 - 1.43)	0.176
125	rs6913578_C_A	0.404	0.398	0.97 (0.76 - 1.24)	0.854	0.395	0.96 (0.78 - 1.19)	0.746
126	rs60954078_G_A	0.361	0.351	0.96 (0.75 - 1.23)	0.754	0.354	0.97 (0.78 - 1.21)	0.825
127	rs57589542_C_CAAAAAAA	0.403	0.397	0.98 (0.76 - 1.25)	0.901	0.396	0.97 (0.78 - 1.20)	0.783
128	rs851984_A_G	0.126	0.118	0.93 (0.64 - 1.34)	0.784	0.114	0.89 (0.65 - 1.24)	0.520
129	rs6904031_T_A	0.080	0.071	0.89 (0.56 - 1.40)	0.736	0.066	0.82 (0.54 - 1.24)	0.372
130	rs910416_C_T	0.442	0.456	1.06 (0.84 - 1.34)	0.671	0.454	1.05 (0.85 - 1.29)	0.669
131	rs9364472_G_C	0.395	0.417	1.09 (0.86 - 1.40)	0.492	0.432	1.17 (0.94 - 1.44)	0.155
132	rs6940159_C_T	0.178	0.181	1.02 (0.75 - 1.39)	0.875	0.190	1.09 (0.83 - 1.42)	0.534
133	rs7971_G_A	0.182	0.199	1.12 (0.83 - 1.51)	0.484	0.185	1.02 (0.78 - 1.34)	0.891
134	rs289997_T_C	0.219	0.231	1.07 (0.81 - 1.42)	0.660	0.224	1.03 (0.80 - 1.33)	0.797
135	rs74765302_A_G	0.002	0	-	1.000	0	-	1.000
136	rs13244925_A_C	0.313	0.317	1.02 (0.79 - 1.31)	0.897	0.313	1.00 (0.80 - 1.25)	1.000
137	rs10644978_ATT_A	0.178	0.171	0.95 (0.69 - 1.30)	0.813	0.167	0.92 (0.70 - 1.22)	0.578
138	rs17268829_C_T	0.268	0.276	1.04 (0.80 - 1.36)	0.786	0.281	1.07 (0.84 - 1.34)	0.591

139	rs4439053_A_G	0.184	0.189	1.04 (0.77 - 1.41)	0.816	0.187	1.02 (0.78 - 1.34)	0.891
140	rs111963714_G_T	0.054	0.040	0.73 (0.40 - 1.32)	0.344	0.043	0.79 (0.48 - 1.30)	0.405
141	rs71559437_A_G	0.077	0.109	1.45 (0.98 - 2.14)	0.062	0.107	1.43 (1.01 - 2.02)	0.044
142	rs7800548_T_C	0.393	0.348	0.82 (0.64 - 1.05)	0.137	0.338	0.79 (0.63 - 0.98)	0.033
143	rs12706954_T_C	0.287	0.300	1.07 (0.82 - 1.38)	0.640	0.303	1.08 (0.86 - 1.36)	0.519
144	rs68056147_A_G	0.317	0.286	0.86 (0.66 - 1.12)	0.269	0.301	0.93 (0.74 - 1.16)	0.530
145	rs5887960_CT_C	0.436	0.375	0.78 (0.61 - 0.99)	0.044	0.389	0.82 (0.67 - 1.02)	0.076
146	rs62485509_T_G	0.048	0.065	1.38 (0.85 - 2.26)	0.214	0.059	1.25 (0.80 - 1.96)	0.331
147	rs66823261_C_T	0.199	0.220	1.14 (0.86 - 1.52)	0.368	0.206	1.05 (0.81 - 1.36)	0.740
148	rs3988353_C_CT	0.330	0.323	0.97 (0.75 - 1.25)	0.846	0.322	0.96 (0.77 - 1.21)	0.775
149	rs1028016_A_G	0.181	0.155	0.83 (0.60 - 1.15)	0.304	0.146	0.77 (0.58 - 1.03)	0.093
150	rs310295_A_C	0.350	0.329	0.91 (0.71 - 1.17)	0.486	0.317	0.86 (0.69 - 1.08)	0.199
151	rs9693444_A_C	0.288	0.270	0.92 (0.70 - 1.19)	0.548	0.265	0.89 (0.70 - 1.13)	0.347
152	rs13365225_G_A	0.301	0.323	1.11 (0.86 - 1.43)	0.432	0.333	1.16 (0.93 - 1.45)	0.206
153	rs1511243_A_G	0.028	0.016	0.55 (0.22 - 1.39)	0.256	0.018	0.65 (0.31 - 1.38)	0.318
154	rs72658084_T_C	0.003	0	-	0.601	0	-	0.602
155	rs1533366_T_G	0.266	0.258	0.96 (0.73 - 1.25)	0.785	0.263	0.98 (0.78 - 1.24)	0.904
156	rs62517052_C_T	0.015	0.009	0.62 (0.19 - 2.05)	0.610	0.007	0.45 (0.14 - 1.50)	0.249
157	rs12546444_T_A	0.124	0.124	1.00 (0.70 - 1.44)	1.000	0.123	0.99 (0.72 - 1.36)	1.000
158	rs13267382_G_A	0.464	0.525	1.28 (1.01 - 1.62)	0.046	0.500	1.16 (0.94 - 1.42)	0.184
159	rs62526620_G_A	0.041	0.043	1.07 (0.60 - 1.92)	0.762	0.048	1.19 (0.72 - 1.95)	0.508
160	rs35542655_C_T	0.192	0.183	0.94 (0.69 - 1.28)	0.759	0.171	0.87 (0.66 - 1.14)	0.342
161	rs12541094_A_G	0.373	0.388	1.07 (0.84 - 1.36)	0.618	0.395	1.10 (0.89 - 1.36)	0.412
162	rs7842619_G_T	0.293	0.209	0.64 (0.48 - 0.85)	0.002	0.229	0.72 (0.56 - 0.92)	0.008
163	rs35961416_CA_C	0.090	0.097	1.10 (0.73 - 1.64)	0.673	0.086	0.95 (0.65 - 1.38)	0.851
164	rs12550713_G_C	0.496	0.491	0.98 (0.77 - 1.24)	0.857	0.484	0.95 (0.77 - 1.17)	0.671
165	rs10096351_A_G	0.258	0.264	1.03 (0.79 - 1.35)	0.836	0.267	1.05 (0.83 - 1.33)	0.717
166	rs1016578_A_G	0.195	0.202	1.04 (0.78 - 1.40)	0.762	0.217	1.14 (0.89 - 1.48)	0.319
167	rs7830152_A_G	0.174	0.150	0.84 (0.60 - 1.17)	0.334	0.151	0.85 (0.64 - 1.13)	0.288
168	rs10975870_G_A	0.006	0	-	0.234	0.002	0.35 (0.05 - 2.73)	0.483
169	rs3057314_C_CAAAA	0.247	0.202	0.77 (0.57 - 1.03)	0.090	0.217	0.84 (0.66 - 1.08)	0.193
170	rs17694493_G_C	0.025	0.025	1.01 (0.47 - 2.16)	1.000	0.021	0.83 (0.40 - 1.71)	0.729
171	rs4880038_T_C	0.294	0.273	0.90 (0.69 - 1.18)	0.466	0.299	1.02 (0.82 - 1.29)	0.861
172	rs665889_T_C	0.198	0.180	0.89 (0.65 - 1.21)	0.494	0.171	0.84 (0.64 - 1.10)	0.226
173	rs10120432_C_T	0.320	0.320	1.00 (0.77 - 1.29)	1.000	0.340	1.09 (0.88 - 1.36)	0.428
174	rs60037937_T_TAA	0.438	0.469	1.14 (0.90 - 1.44)	0.302	0.443	1.02 (0.83 - 1.26)	0.873
175	rs10816625_G_A	0.366	0.373	1.03 (0.81 - 1.31)	0.851	0.361	0.98 (0.79 - 1.21)	0.869
176	rs13294895_T_C	0.026	0.016	0.59 (0.23 - 1.48)	0.331	0.021	0.78 (0.38 - 1.60)	0.612
177	rs7848334_G_T	0.015	0.006	0.40 (0.09 - 1.67)	0.303	0.016	1.03 (0.45 - 2.37)	1.000
178	rs630965_C_T	0.073	0.068	0.93 (0.58 - 1.48)	0.817	0.062	0.83 (0.54 - 1.27)	0.469
179	rs1895062_G_A	0.289	0.317	1.14 (0.89 - 1.48)	0.320	0.310	1.11 (0.89 - 1.39)	0.382
180	rs3861871_A_G	0.423	0.475	1.24 (0.97 - 1.57)	0.086	0.484	1.28 (1.04 - 1.58)	0.023
181	9:136146597:C:T_T_C	0.188	0.168	0.87 (0.64 - 1.20)	0.437	0.192	1.03 (0.79 - 1.34)	0.839
182	c10_pos5834658_G_A	0.146	0.130	0.88 (0.62 - 1.25)	0.493	0.135	0.91 (0.67 - 1.23)	0.597
183	rs10796139_G_A	0.369	0.394	1.12 (0.88 - 1.42)	0.383	0.395	1.12 (0.90 - 1.38)	0.323
184	rs7072776_A_G	0.071	0.062	0.87 (0.53 - 1.41)	0.636	0.071	1.00 (0.66 - 1.49)	1.000
185	10:22477776:ACC:A_A_ACC	0.011	0.006	0.58 (0.13 - 2.49)	0.760	0.016	1.50 (0.63 - 3.58)	0.329
186	rs10764337_A_C	0.063	0.053	0.84 (0.50 - 1.41)	0.614	0.059	0.95 (0.61 - 1.47)	0.912
187	rs2384736_C_A	0.460	0.463	1.01 (0.80 - 1.28)	0.952	0.473	1.05 (0.85 - 1.30)	0.632
188	rs10995201_G_A	0.022	0.012	0.56 (0.20 - 1.58)	0.392	0.014	0.62 (0.26 - 1.47)	0.348
189	rs6479868_T_G	0.154	0.143	0.91 (0.65 - 1.28)	0.675	0.153	0.99 (0.74 - 1.32)	1.000
190	rs111833376_T_C	0.110	0.084	0.74 (0.49 - 1.12)	0.171	0.082	0.72 (0.50 - 1.05)	0.099
191	rs719338_G_T	0.400	0.407	1.03 (0.81 - 1.31)	0.854	0.420	1.09 (0.88 - 1.34)	0.449
192	rs4980029_G_A	0.449	0.432	0.93 (0.73 - 1.18)	0.585	0.441	0.97 (0.78 - 1.19)	0.749
193	rs140936696_CAA_C	0.328	0.331	1.01 (0.79 - 1.30)	0.949	0.337	1.04 (0.83 - 1.30)	0.734
194	rs10885405_T_C	0.050	0.028	0.55 (0.27 - 1.10)	0.113	0.032	0.63 (0.36 - 1.12)	0.129
195	rs12250948_C_T	0.223	0.248	1.15 (0.87 - 1.51)	0.314	0.237	1.08 (0.85 - 1.39)	0.526
196	rs9421410_A_G	0.383	0.391	1.04 (0.81 - 1.32)	0.804	0.384	1.00 (0.81 - 1.24)	1.000
197	rs45631580_G_A	0.114	0.112	0.98 (0.67 - 1.43)	1.000	0.112	0.98 (0.71 - 1.36)	1.000
198	rs35054928_GC_G	0.483	0.463	0.92 (0.73 - 1.17)	0.507	0.489	1.02 (0.83 - 1.26)	0.874
199	rs45631563_T_A	0.013	0.003	0.23 (0.03 - 1.70)	0.162	0.002	0.17 (0.02 - 1.25)	0.045
200	rs7394715_T_C	0.003	0	-	1.000	0.002	0.85 (0.10 - 7.32)	1.000
201	rs6597981_G_A	0.242	0.230	0.93 (0.71 - 1.24)	0.672	0.237	0.98 (0.76 - 1.25)	0.901
202	rs4980386_C_A	0.330	0.335	1.03 (0.80 - 1.32)	0.848	0.342	1.06 (0.85 - 1.32)	0.612
203	rs10832963_T_G	0.466	0.494	1.12 (0.88 - 1.42)	0.364	0.477	1.04 (0.85 - 1.29)	0.709
204	rs4472923_T_C	0.239	0.239	1.00 (0.76 - 1.32)	1.000	0.258	1.10 (0.87 - 1.40)	0.421
205	rs10838267_A_G	0.324	0.353	1.14 (0.89 - 1.46)	0.303	0.328	1.02 (0.82 - 1.27)	0.865
206	rs77047825_G_C	0.001	0	-	1.000	0	-	1.000
207	rs12287832_A_C	0.108	0.096	0.88 (0.59 - 1.31)	0.624	0.116	1.09 (0.79 - 1.51)	0.610
208	rs10896047_A_G	0.240	0.261	1.12 (0.85 - 1.47)	0.439	0.251	1.06 (0.84 - 1.35)	0.620
209	rs35039974_T_A	0.190	0.244	1.37 (1.04 - 1.81)	0.033	0.222	1.22 (0.94 - 1.57)	0.141
210	rs661204_A_G	0.033	0.019	0.55 (0.24 - 1.29)	0.221	0.027	0.82 (0.44 - 1.54)	0.651
211	rs78540526_T_C	0.017	0.006	0.37 (0.09 - 1.56)	0.215	0.011	0.69 (0.27 - 1.77)	0.526
212	rs7125780_T_G	0.367	0.323	0.82 (0.64 - 1.06)	0.132	0.336	0.87 (0.70 - 1.08)	0.225
213	rs199504893_CA_C	0.398	0.419	1.09 (0.86 - 1.39)	0.499	0.425	1.11 (0.90 - 1.38)	0.330
214	rs610437_T_C	0.278	0.242	0.83 (0.63 - 1.09)	0.198	0.247	0.85 (0.67 - 1.08)	0.190
215	rs625145_T_A	0.180	0.174	0.96 (0.70 - 1.31)	0.875	0.180	1.01 (0.77 - 1.32)	0.945
216	rs7121616_G_A	0.336	0.357	1.09 (0.86 - 1.40)	0.484	0.354	1.08 (0.87 - 1.34)	0.501

217	rs7939702_T_G	0.029	0.037	1.30 (0.69 - 2.46)	0.381	0.037	1.28 (0.72 - 2.25)	0.438
218	rs11822830_A_G	0.472	0.407	0.77 (0.60 - 0.98)	0.034	0.413	0.79 (0.64 - 0.97)	0.029
219	12:293626:A:G_G_A	0.438	0.435	0.99 (0.78 - 1.25)	0.952	0.416	0.91 (0.74 - 1.13)	0.422
220	rs12422552_C_G	0.306	0.286	0.91 (0.70 - 1.18)	0.511	0.310	1.02 (0.82 - 1.28)	0.863
221	rs788458_T_C	0.183	0.162	0.86 (0.63 - 1.19)	0.388	0.169	0.91 (0.69 - 1.20)	0.534
222	rs7297051_T_C	0.217	0.208	0.95 (0.71 - 1.26)	0.769	0.215	0.98 (0.76 - 1.27)	0.949
223	12:28347382:C:T_T_C	0.021	0.022	1.04 (0.46 - 2.35)	0.835	0.018	0.87 (0.41 - 1.88)	0.852
224	12:29140260:G:A_G_A	0.209	0.239	1.19 (0.90 - 1.57)	0.239	0.251	1.27 (0.99 - 1.61)	0.062
225	rs2277339_G_T	0.173	0.143	0.80 (0.57 - 1.11)	0.198	0.155	0.88 (0.66 - 1.17)	0.397
226	12:70798355:A:T_T_A	0.368	0.357	0.96 (0.75 - 1.22)	0.754	0.345	0.91 (0.73 - 1.13)	0.378
227	12:83064195:G:GA_GA_G	0.178	0.177	0.99 (0.73 - 1.35)	1.000	0.178	1.00 (0.76 - 1.31)	1.000
228	12:85004551:C:T_T_C	0.116	0.134	1.18 (0.83 - 1.68)	0.350	0.142	1.26 (0.93 - 1.71)	0.142
229	rs17356907_G_A	0.241	0.224	0.91 (0.68 - 1.20)	0.525	0.226	0.92 (0.72 - 1.18)	0.534
230	12:103097887:C:T_T_C	0.016	0.009	0.60 (0.18 - 1.97)	0.613	0.009	0.59 (0.20 - 1.67)	0.378
231	12:111600134:G:T_T_G	0.008	0.006	0.77 (0.18 - 3.40)	1.000	0.007	0.85 (0.25 - 2.96)	1.000
232	12:115108136:T:C_C_T	0.469	0.497	1.12 (0.88 - 1.42)	0.365	0.509	1.18 (0.96 - 1.45)	0.137
233	12:115796577:A:G_G_A	0.267	0.255	0.94 (0.72 - 1.23)	0.682	0.272	1.02 (0.81 - 1.29)	0.857
234	rs2454399_C_T	0.251	0.242	0.95 (0.72 - 1.26)	0.781	0.237	0.93 (0.73 - 1.19)	0.581
235	12:120832146:C:T_T_C	0.081	0.056	0.67 (0.40 - 1.11)	0.142	0.064	0.77 (0.51 - 1.17)	0.276
236	rs56404467_A_G	0.002	0	-	1.000	0	-	1.000
237	rs11571833_T_A	0.001	0	-	1.000	0	-	1.000
238	rs9315973_A_G	0.388	0.403	1.07 (0.84 - 1.36)	0.620	0.431	1.20 (0.97 - 1.48)	0.103
239	rs12870942_C_T	0.277	0.242	0.83 (0.63 - 1.10)	0.198	0.242	0.83 (0.65 - 1.06)	0.152
240	rs2181965_A_G	0.020	0.016	0.78 (0.31 - 2.00)	0.825	0.014	0.69 (0.29 - 1.64)	0.555
241	rs34914085_A_C	0.272	0.276	1.02 (0.78 - 1.33)	0.892	0.260	0.94 (0.74 - 1.19)	0.675
242	rs2253012_T_C	0.129	0.106	0.80 (0.54 - 1.16)	0.275	0.103	0.77 (0.55 - 1.08)	0.147
243	rs2588809_T_C	0.032	0.050	1.58 (0.90 - 2.78)	0.135	0.039	1.22 (0.70 - 2.11)	0.461
244	rs11624333_C_T	0.051	0.040	0.79 (0.44 - 1.42)	0.488	0.034	0.66 (0.38 - 1.16)	0.170
245	rs11341843_T_TC	0.449	0.429	0.92 (0.72 - 1.17)	0.505	0.464	1.06 (0.86 - 1.30)	0.594
246	rs941764_G_A	0.153	0.146	0.95 (0.68 - 1.32)	0.801	0.142	0.91 (0.68 - 1.23)	0.604
247	rs78440108_T_C	0.014	0.019	1.30 (0.53 - 3.17)	0.617	0.016	1.11 (0.48 - 2.56)	0.825
248	rs4983544_T_G	0.345	0.275	0.72 (0.55 - 0.94)	0.015	0.278	0.73 (0.58 - 0.92)	0.008
249	rs187010898_A_C	0	0	-	1.000	0	-	1.000
250	rs4774565_G_A	0.483	0.491	1.03 (0.81 - 1.31)	0.808	0.488	1.02 (0.83 - 1.26)	0.872
251	rs8042593_G_A	0.238	0.233	0.97 (0.73 - 1.28)	0.887	0.219	0.90 (0.70 - 1.15)	0.416
252	rs35874463_G_A	0.003	0	-	1.000	0	-	0.591
253	rs8035987_C_T	0.355	0.366	1.05 (0.82 - 1.35)	0.703	0.350	0.98 (0.78 - 1.22)	0.866
254	rs2290202_T_G	0.447	0.435	0.95 (0.75 - 1.21)	0.716	0.468	1.09 (0.88 - 1.34)	0.455
255	rs144767203_C_A	0.218	0.248	1.19 (0.90 - 1.57)	0.218	0.233	1.09 (0.85 - 1.40)	0.482
256	rs57920543_CAAAAA_C	0.059	0.063	1.07 (0.66 - 1.75)	0.797	0.062	1.06 (0.69 - 1.64)	0.822
257	rs11076805_A_C	0.106	0.087	0.80 (0.53 - 1.21)	0.323	0.100	0.94 (0.67 - 1.32)	0.795
258	rs12709163_C_G	0.115	0.149	1.35 (0.96 - 1.89)	0.094	0.151	1.37 (1.01 - 1.84)	0.043
259	rs34872983_A_G	0.264	0.298	1.18 (0.91 - 1.53)	0.221	0.308	1.24 (0.99 - 1.56)	0.065
260	rs75753503_T_G	0.019	0.019	1.00 (0.42 - 2.39)	1.000	0.018	0.98 (0.45 - 2.12)	1.000
261	rs35668161_A_C	0.203	0.224	1.13 (0.85 - 1.51)	0.412	0.222	1.12 (0.87 - 1.44)	0.394
262	rs4784227_T_C	0.285	0.304	1.10 (0.85 - 1.42)	0.464	0.301	1.08 (0.86 - 1.36)	0.482
263	rs55872725_T_C	0.167	0.168	1.01 (0.73 - 1.38)	0.936	0.174	1.05 (0.80 - 1.38)	0.723
264	rs6499648_C_T	0.296	0.360	1.34 (1.04 - 1.72)	0.022	0.345	1.25 (1.00 - 1.56)	0.050
265	rs7184573_A_G	0.198	0.186	0.93 (0.69 - 1.26)	0.704	0.176	0.87 (0.66 - 1.14)	0.314
266	rs28539243_G_A	0.381	0.379	0.99 (0.78 - 1.26)	0.951	0.379	0.99 (0.80 - 1.23)	0.956
267	rs7500067_G_A	0.252	0.289	1.21 (0.93 - 1.57)	0.167	0.278	1.15 (0.91 - 1.45)	0.249
268	rs9931038_C_T	0.172	0.158	0.91 (0.66 - 1.25)	0.575	0.155	0.88 (0.67 - 1.18)	0.436
269	rs12449271_C_T	0.175	0.183	1.06 (0.78 - 1.44)	0.751	0.174	0.99 (0.75 - 1.30)	1.000
270	rs79461387_T_G	0.112	0.090	0.79 (0.52 - 1.18)	0.286	0.100	0.89 (0.63 - 1.25)	0.552
271	rs150537328_C_T	0.005	0.003	0.64 (0.08 - 5.11)	1.000	0.002	0.47 (0.06 - 3.75)	0.698
272	rs11296_C_T	0	0	-	1.000	0	-	1.000
273	rs17881320_T_G	0.005	0	-	0.375	0.002	0.43 (0.05 - 3.34)	0.701
274	rs149370081_A_G	0	0	-	1.000	0	-	1.000
275	rs71363517_CT_C	0.109	0.112	1.03 (0.71 - 1.50)	0.847	0.098	0.89 (0.63 - 1.26)	0.548
276	17:44283858:G:A_A_G	0.005	0	-	0.373	0.005	0.95 (0.20 - 4.41)	1.000
277	rs2787486_C_A	0.293	0.298	1.03 (0.79 - 1.33)	0.843	0.297	1.02 (0.81 - 1.28)	0.861
278	rs745570_G_A	0.433	0.435	1.01 (0.79 - 1.28)	0.952	0.420	0.95 (0.77 - 1.17)	0.668
279	rs16976596_T_C	0.025	0.012	0.50 (0.18 - 1.40)	0.226	0.014	0.55 (0.23 - 1.30)	0.210
280	rs11665269_T_C	0.441	0.388	0.81 (0.63 - 1.03)	0.088	0.411	0.89 (0.72 - 1.09)	0.262
281	rs1111207_T_C	0.332	0.311	0.91 (0.70 - 1.17)	0.480	0.313	0.92 (0.73 - 1.15)	0.463
282	rs527616_C_G	0.250	0.205	0.78 (0.58 - 1.04)	0.091	0.210	0.80 (0.62 - 1.03)	0.094
283	rs35369219_A_AT	0.465	0.516	1.22 (0.97 - 1.55)	0.102	0.511	1.20 (0.98 - 1.48)	0.088
284	rs8092192_G_C	0.495	0.481	0.95 (0.75 - 1.20)	0.673	0.491	0.98 (0.80 - 1.21)	0.874
285	rs72931898_A_G	0.015	0.009	0.60 (0.18 - 1.97)	0.613	0.007	0.44 (0.13 - 1.45)	0.253
286	rs9954058_C_G	0.103	0.115	1.13 (0.78 - 1.64)	0.554	0.098	0.95 (0.67 - 1.34)	0.793
287	rs9952980_C_T	0.331	0.314	0.93 (0.72 - 1.19)	0.564	0.315	0.93 (0.74 - 1.16)	0.572
288	rs117922601_T_G	0.001	0	-	1.000	0	-	1.000
289	rs56069439_A_C	0.011	0.003	0.27 (0.04 - 2.05)	0.235	0.005	0.40 (0.09 - 1.73)	0.288
290	rs10164323_T_C	0.228	0.248	1.12 (0.85 - 1.48)	0.431	0.247	1.11 (0.87 - 1.42)	0.413
291	rs140702307_CGGGCG_C	0.438	0.444	1.03 (0.81 - 1.30)	0.855	0.434	0.98 (0.80 - 1.22)	0.915
292	rs56681946_C_T	0.159	0.158	0.99 (0.72 - 1.37)	1.000	0.162	1.02 (0.77 - 1.36)	0.885
293	rs4399645_C_T	0.423	0.453	1.13 (0.89 - 1.44)	0.329	0.425	1.01 (0.82 - 1.24)	0.957
294	rs1172821_T_C	0.128	0.127	0.99 (0.70 - 1.41)	1.000	0.124	0.96 (0.70 - 1.32)	0.873

295	rs16991615_A_G	0.006	0	-	0.385	0	-	0.237
296	rs1154723_T_C	0.002	0.003	1.46 (0.16 - 13.06)	0.548	0.002	1.07 (0.12 - 9.59)	1.000
297	rs6030585_C_G	0.160	0.177	1.13 (0.83 - 1.55)	0.461	0.171	1.09 (0.82 - 1.44)	0.565
298	rs13039563_A_G	0.358	0.363	1.02 (0.80 - 1.31)	0.900	0.369	1.05 (0.84 - 1.30)	0.698
299	rs2822999_G_T	0.145	0.127	0.86 (0.61 - 1.23)	0.438	0.144	0.99 (0.74 - 1.33)	1.000
300	rs2823130_G_A	0.110	0.118	1.08 (0.75 - 1.56)	0.701	0.105	0.95 (0.68 - 1.33)	0.799
301	rs2403907_A_C	0.105	0.102	0.97 (0.66 - 1.43)	1.000	0.114	1.10 (0.79 - 1.52)	0.606
302	rs4818836_A_G	0.006	0	-	0.385	0	-	0.237
303	rs9798754_C_T	0.437	0.460	1.10 (0.86 - 1.39)	0.466	0.454	1.07 (0.87 - 1.32)	0.521
304	rs17879961_G_A	0	0	-	1.000	0	-	1.000
305	rs5997390_A_G	0.091	0.078	0.84 (0.54 - 1.30)	0.524	0.089	0.98 (0.68 - 1.41)	1.000
306	rs34134147_T_C	0.001	0	-	1.000	0	-	1.000
307	rs132289_A_G	0	0	-	1.000	0	-	1.000
308	rs373038216_AAAAGAAAG _AAAAG	0.263	0.266	1.01 (0.78 - 1.33)	0.945	0.252	0.95 (0.74 - 1.20)	0.672
309	rs5750715_T_A	0.468	0.447	0.92 (0.73 - 1.17)	0.506	0.466	0.99 (0.80 - 1.22)	0.958
310	rs66987842_C_CT	0.197	0.181	0.90 (0.67 - 1.23)	0.593	0.184	0.92 (0.70 - 1.20)	0.591
311	rs9611990_T_C	0.006	0.006	0.97 (0.22 - 4.35)	1.000	0.007	1.07 (0.30 - 3.80)	1.000
312	rs112855987_A_G	0.254	0.214	0.80 (0.60 - 1.07)	0.142	0.242	0.94 (0.74 - 1.20)	0.625
313	rs28512361_A_G	0.040	0.044	1.10 (0.61 - 1.97)	0.758	0.039	0.98 (0.57 - 1.67)	1.000

Supplementary Table S4. Association between 313-single nucleotide polymorphism (SNP) breast cancer polygenic risk score and neutropenia-related outcomes in chemotherapy-treated breast cancer patients who did not receive granulocyte colony-stimulating factor (G-CSF). Controls were chemotherapy-treated breast cancer patients who did not develop neutropenia. [1]Adjusted for recruitment site, ethnicity, BMI, population structure (first four principal components), tumour size, tumour grade, and estrogen-receptor status. FNc: febrile neutropenia from initiation of chemotherapy treatment (using taxanes or anthracyclines) to 30 days from last chemotherapy treatment cycle (i.e. within 30 days of last chemotherapy treatment); OR: odds ratio; CI: confidence interval; SNP: single nucleotide polymorphism; PRS: polygenic risk score; $PRS_{overall}$: Overall PRS; ER: Estrogen receptor; $PRS_{ER\text{-}pos}$: ER-positive PRS; $PRS_{ER\text{-}neg}$: ER-negative PRS.

	**FNc n=161**				**Neutropenia n=219**			
	**Crude**		**Adjusted[1]**		**Crude**		**Adjusted[1]**	
	**OR (95% CI)**	***p*-value**	**OR (95% CI)**	***p*-value**	**OR (95% CI)**	***p*-value**	**OR (95% CI)**	***p*-value**
**313-SNP breast cancer polygenic risk score**								
**$PRS_{overall}$**								
Quartile 1	1.00 (Reference)		1.00 (Reference)		1.00 (Reference)		1.00 (Reference)	
Quartile 2	0.92 (0.59 - 1.45)	0.723	0.94 (0.55 - 1.61)	0.834	1.04 (0.69 - 1.55)	0.863	1.05 (0.66 - 1.68)	0.822
Quartile 3	0.84 (0.54 - 1.33)	0.462	0.84 (0.49 - 1.42)	0.507	0.84 (0.56 - 1.27)	0.412	0.82 (0.51 - 1.31)	0.410
Quartile 4	0.64 (0.39 - 1.05)	0.079	0.61 (0.35 - 1.08)	0.089	0.78 (0.51 - 1.19)	0.243	0.79 (0.49 - 1.28)	0.336
Continuous	0.87 (0.73 - 1.03)	0.097	0.85 (0.70 - 1.04)	0.111	0.91 (0.79 - 1.06)	0.235	0.91 (0.77 - 1.08)	0.272
**$PRS_{ER\text{-}pos}$**								
Quartile 1	1.00 (Reference)		1.00 (Reference)		1.00 (Reference)		1.00 (Reference)	
Quartile 2	1.14 (0.72 - 1.79)	0.583	1.28 (0.75 - 2.21)	0.365	1.24 (0.83 - 1.87)	0.291	1.41 (0.88 - 2.25)	0.154
Quartile 3	0.94 (0.59 - 1.50)	0.800	0.95 (0.56 - 1.62)	0.847	0.93 (0.61 - 1.42)	0.749	0.96 (0.60 - 1.55)	0.881
Quartile 4	0.77 (0.47 - 1.26)	0.305	0.76 (0.43 - 1.35)	0.354	0.93 (0.60 - 1.42)	0.722	0.96 (0.59 - 1.55)	0.862
Continuous	0.87 (0.74 - 1.03)	0.105	0.86 (0.71 - 1.04)	0.119	0.93 (0.80 - 1.08)	0.320	0.92 (0.78 - 1.09)	0.358
**$PRS_{ER\text{-}neg}$**								
Quartile 1	1.00 (Reference)		1.00 (Reference)		1.00 (Reference)		1.00 (Reference)	
Quartile 2	0.58 (0.36 - 0.93)	0.025	0.56 (0.32 - 0.97)	0.039	0.61 (0.40 - 0.92)	0.019	0.59 (0.36 - 0.95)	0.029
Quartile 3	0.54 (0.34 - 0.87)	0.012	0.53 (0.31 - 0.91)	0.022	0.60 (0.40 - 0.91)	0.017	0.60 (0.37 - 0.95)	0.031
Quartile 4	0.71 (0.46 - 1.10)	0.129	0.67 (0.40 - 1.12)	0.126	0.72 (0.49 - 1.07)	0.102	0.71 (0.46 - 1.11)	0.133
Continuous	0.86 (0.73 - 1.02)	0.089	0.86 (0.71 - 1.04)	0.120	0.86 (0.74 - 1.00)	0.045	0.85 (0.72 - 1.01)	0.064
